# Biofilm-Inhibitory Activity of Wild Mushroom Extracts against Pathogenic Bacteria

**DOI:** 10.1155/2024/7011982

**Published:** 2024-01-29

**Authors:** Gebreselema Gebreyohannes, Desta Berhe Sbhatu, Andrew Kimang'a Nyerere, Abrha Gebreselema Gebrehiwot

**Affiliations:** ^1^Department of Biological and Chemical Engineering, Mekelle Institute of Technology, Mekelle University, Mekele, Ethiopia; ^2^Department of Medical Microbiology, College of Health Sciences, Jomo Kenyatta University of Agriculture and Technology, Nairobi, Kenya; ^3^Department of Medical Biochemistry, College of Health Sciences, Mekelle University, Mekele, Ethiopia

## Abstract

**Objective:**

This study aims to investigate the bacterial biofilm-inhibitory effect of mushroom extracts.

**Methods:**

Mushrooms were collected from Arabuko-Sokoke and Kakamega forests and identified using morphological and molecular approaches. *Auricularia auricula-judae*, *Microporus xanthopus*, *Termitomyces umkowaani*, *Trametes elegans*, and *Trametes versicolor* were extracted by chloroform, 70% ethanol, and hot water. Extracts were tested against *Escherichia coli*, *Pseudomonas aeruginosa*, and *Staphylococcus aureus* (ATCC25923). Data were analyzed using SPSS ver. 20.0.

**Results:**

Chloroform, 70% ethanol, and hot water extracts of *A. auricula-judae* (50 *μ*g/mL) showed statistically significant antibiofilm activities against *P. aeruginosa*, *E. coli*, and *S. aureus* (*p* ≤ 0.05). *M. xanthopus* extracts (250 *μ*g/mL) revealed significantly significant antibiofilm activities against each test bacterium (*p* ≤ 0.05). All extracts of *T. umkowaani* (250 *μ*g/mL) exhibited statistically significant antibiofilm activities against *S. aureus* only (*p* ≤ 0.05). Chloroform extract of *T. elegans* (250 *μ*g/mL) showed the best antibiofilm activity (69.75 ± 0.01%) against *S. aureus.* All *T. versicolor* extracts (250 *μ*g/mL) indicated the best antibiofilm activities against *S. aureus*.

**Conclusions:**

Being the first study of its kind to be conducted in Kenya, it added a novel concept to the body of knowledge already known about medical biotechnology research. It offers a fresh understanding of the various varieties of mushrooms found in Kenya, their potential biological function in the production of drugs, particularly those that combat drug resistance, and perhaps even a peek at their bioactive elements. Wild mushrooms, a hidden gem, might help to reopen the pipeline of new antibiotics that have been on the decline. However, further research is required to determine the potential mechanism(s) of action of the extracts that are in charge of the apparent antibiofilm activity.

## 1. Introduction

Bacterial biofilms are collections of bacterial cells that are adhered to surfaces and/or to one another and enclosed in an extracellular matrix that the bacteria have created on their own [1, 2]. Bacteria that produce biofilms are extremely resistant to antimicrobial treatments [3, 4]. The damaging effects of antibacterial agents and environmental stresses are warded off by bacterial biofilm. Due to the fact that most antibiotics are unable to pass through the biofilm's protective coating, it has a higher resistance (10–100 times) than the planktonic bacterial cells it is made of [1, 5]. Understanding biofilms' secret and how they develop antimicrobial resistance has recently drawn more interest from researchers [6]. The creation of new therapeutic approaches is necessary given the quick dissemination and ongoing generation of infections and biofilms that are resistant to antibiotics [7, 8]. The need for alternatives is being fueled by the growing disparity between the advent of bacteria resistant to antibiotics and the paucity of newly developed therapeutic methods. The search for effective screening and finding promising antibiofilm chemicals from natural sources is constantly expanding [9–11]. Studies have been concentrating on natural secondary metabolites acting as nontoxic inhibitors of the quorum-sensing process within the biofilms to control infections with no encouraging findings against resistant strains [12].

The successful prevention and efficient treatment of bacterial infections are currently threatened globally by antibiotic resistance [13–15]. Clinical and therapeutic results have been negatively impacted by antibiotic resistance, with effects ranging from treatment failures to greater rates of morbidity and mortality as well as high healthcare expenditures [16]. Despite the fact that numerous steps have been taken in recent years to address this problem, the trend of worldwide antimicrobial resistance has not changed as of yet. The primary causes of the emergence of antimicrobial resistance are the inappropriate and excessive use of various antibacterial agents for various objectives. Target alteration, extensive antibiotics-degrading enzyme production, porin alteration, efflux pump overexpression, spontaneous evolution, bacterial mutation, and horizontal gene transfer are other important factors in the development of antimicrobial resistance [13, 17]. The need for novel antibiotics and other antimicrobials is still urgent in the fight against bacterial infections by humans [14]. Antibiotic-resistance catastrophe is looming due to this worrying confluence of increasing antibiotic resistance and declining innovation. In the worst-case scenario, most bacterial illnesses would once again be resistant to therapeutic treatment, sending humanity back to the preantibiotic age [15]. Since natural products are a significant source of chemical variety and have given significant therapeutic agents for many bacterial infections, there has been a rise in interest in the mechanisms behind their actions in recent years. Mushrooms have long been valued for their renowned source of products with a variety of bioactivities, from antibacterial to antiviral, cytotoxic, anti-inflammatory, antifungal, or antioxidant, and they may be a useful source in the search for new bioactive extracts to inhibit biofilm production [18]. Giving researchers more money will speed up and expand screening campaigns, significantly increasing the chance that an antibiotic will be discovered [15].

Fungal species are already providing a wide range of potential for bioprospecting and downstream applications [19]. To address issues with biofilm development, systematic exploration and evaluation of bioactive substances from wild mushrooms are thought to be quite beneficial [16, 20]. Finding and analysing bioactive components in mushrooms are highly beneficial for reducing the growth of biofilms [21]. In this regard, the identification of antibiofilm chemicals in wild mushrooms may herald a new era in the fight against microbial disease through the development of more effective methods for dealing with drug-resistant microorganisms [22]. However, no research has been done to date to look for antibiofilm bioactive components in these five wild mushrooms from the Kenyan woodlands of Arabuko-Sokoke and Kakamega. Therefore, the aim of this work is to investigate the bioactive substances found in various wild mushrooms from Kenya by assessing their biofilm-inhibitory effects against three harmful bacteria.

## 2. Materials and Methods

### 2.1. Wild Mushrooms Collection

The wild mushrooms growing on tree barks and other substrates (wood, soil, and leaf litter) were randomly collected and handled carefully, wrapped in aluminum foil, and transported to the laboratory.

### 2.2. Morphological and Molecular Identification of Specimens

Specimens were identified using spore print color (i.e., white, black, brown, pink, and purple) and macroscopic and microscopic (i.e., shape and size of basidiospores, basidia, cystidia, and generative hyphae) methods as well as by comparing their morphological characteristics to *Species Fungorum* and related literature. Morphological parameters of the mushrooms such as the color and shape of the cap; the color and shape of the stipe; and the size and shape of the gill are some of the parameters used for identification. The pileal margin and pileal surface, stipe location, stipe base, and gill margin were also used for the identification. Moreover, other morphological observations of the mushrooms, such as the structure of the cap; margin and location of the gills; location, surface, and shape of the stipe; and margin, shape, and surface of the pileus, were recorded. Furthermore, other characteristics such as the ornamentation of the surfaces of the pileus and stipe, the presence and absence of an annulus on the stipe, and the presence or absence of volva at the base of the stipe were also used to describe and identify the mushrooms (Table1). Finally, specimens were dried in an electric drying oven at 40 to 50°C for 168 h. and were kept for further analyses [23, 24].

Genomic DNA was extracted from the dried fruiting body of mushrooms using the cetyl trimethyl ammonium bromide (CTAB) method [25]. Highly conserved regions of fungal rDNA-specific genes (i.e., ITS1 and ITS4) were amplified using the PCR amplification method [26]. During PCR amplification, ITS1-F (5′-CTT GGT CAT TTA GAG GAA GTA A-3′) and ITS-4 (5′-TCC TCC GCT TAT TGA TAT GC-3′) set of forward and reverse primers were used, respectively (Table 2). The amplification reaction volume (100 *μ*L) contained 2.5 *μ*L of 80–100 ng of specific genes (ITS1 and ITS4), 1.5 *μ*L of sterile Milli Q water, 1 *μ*L of 20 pmol of each primer, and 20 *μ*L of OneTaq Quick-Load 2x master mix (containing 0.25 mM each dNTPs, 2 mM = 0.19 g MgCl_2_, and *Taq* DNA polymerase). Control reactions without the DNA template were also prepared. The PCR was performed in the following conditions: initial denaturation at 94°C for 5 min followed by 30 cycles of denaturation at 94°C for 1 min, annealing for 1 min at 52°C, initial extension for 1 min at 72°C, and final extension of 10 min at 72°C, followed by cooling at 4°C until the samples were recovered. Amplified PCR products were separated using electrophoresis and visualized under UV light. The presence and the amount of each DNA PCR product were estimated by comparing it against the ethidium bromide fluorescence intensity of control of 1 kb DNA ladder.

### 2.3. Extraction of Bioactive Compounds

Bioactive compounds were extracted using chloroform, 70% ethanol, and hot water solvents using previously reported methods [27–30]. A 100 g powder of each mushroom was transferred into three Erlenmeyer flasks. The first flask was mixed with 1.0 L of 99.8% chloroform (Sigma-Aldrich, USA), the second flask was mixed with 70% ethanol (99.9%) (ECP Ltd, New Zealand), and the third flask was mixed with distilled hot water heated at 60°C, and it waited for 2 h. at 25°C. Flasks were shaken for 72 h at 150 rpm using an incubator shaker (SK-727, Amerex Instuments, Inc., USA). The extracts were centrifuged at 3,000 rpm (Eppendorf Centrifuge 5810 R, Germany) for 15 min, filtered with Whatman No. 1 filter paper, and concentrated and dried by a rotary evaporator (EV311, Lab Tech Co. Ltd., UK) at 50°C. The extracts were kept in a −80°C deep freezer and freeze-dried (MRC Freeze Dryer, Model, FDL-10N-50-8M). Finally, crude extracts were stored in a 4°C refrigerator in amber-colored bottles for further analyses.

### 2.4. Study Design for Antibiofilm Activities of Mushroom Extracts

The antibiofilm activities of the extracts were determined by microtiter plate assay [31, 32]. *Escherichia coli* (Clinical isolate), *Pseudomonas aeruginosa* (Clinical isolate), and *Staphylococcusaureus* (ATCC25923) were inoculated to 5 mL Mueller Hinton broth (MHB) test tubes and incubated at 37°C for 18 h. The cultures of each bacterium were diluted 100-fold (1 : 100) with a fresh MHB to obtain OD_620_ of 1.00 (1 × 10^8^ CFU/mL). 20 mg/mL of each mushroom extract was dissolved in dimethyl sulfoxide (DMSO). A sterile 100 *μ*L MHB was added to the wells. From the 20 mg/mL extract, 25 *μ*L (500 *μ*g/mL) was dispensed to the #1 Well of rows A, B, and C of the 96 wells of the microtiter plate. Then, the mixture of the broth and the extract was serially diluted from Well #1 up to Well #4 (the concentration of the extract ranged from 500 *μ*g/mL Well #1 to 62.5 *μ*g/mL Well #4). The wells of the other three rows (rows D, E, and F) within the same microtiter plate were filled with 100 *μ*L of MHB and 100 *μ*L of each bacterium culture without the extracts as a negative control.

The wells of the remaining two rows (rows G and H) were filled with 200 *μ*L Mueller Hinton broth only as quality control. The microtiter plates were incubated at 37°C for 48 h. After incubation, 100 *μ*L of planktonic bacterial cells was transferred to a new 96-well cell culture plate, and the OD_620_ was measured. The antibacterial effects of the extracts on the planktonic cells of the tested bacteria were investigated by calculating the relative amounts of planktonic cells of the tested bacteria and comparing their optical density (OD) values against their counterparts (negative controls). The contents of each well were discarded and washed three times with sterile distilled water to remove nonadherent cells and air-dried for 15 min to visualize the biofilm formation. The microtiter plate wells were fixed using 200 *μ*L of 2% sodium acetate at room temperature for 10 min, and the sodium acetate solution was discarded and air-dried.

A 200 *μ*L of 0.10% crystal violet (CV) solution was added to the wells and waited for 10 min at room temperature, and the excess stain was rinsed off with distilled water. Subsequently, the microtiter plates were vigorously tapped on a paper towel to remove any excess liquid and air-dried. The attached and stained bacterial cells (if any) were solubilized with 200 *μ*L of glacial acetic acid for 15 min at room temperature [33]. About 200 *μ*L of the CV and glacial acetic acid mixed solution in the wells were then transferred to other microtiter plate wells. Biofilm formation (adherence) was quantified by measuring the optical density of the CV and glacial acetic acid mixed solution at 620 nm using a microtiter plate reader (Infinite F50 Wako, TECAN). The percentage of biofilm inhibition was calculated by the formula: [(Control OD_620nm_ − Test OD_620nm_) ÷ Control OD_620nm_] × 100. The amount of biofilm formed was measured by comparing the absorbance values of the extract-treated bacteria against the untreated ones.

### 2.5. Data Analyses

All experiments and tests were performed in triplicates. All quantitative data were compared using relevant descriptive and inferential statistics using SPSS ver. 20.0. Results of data analyses are given as mean of triplicates and standard deviation (mean ± SD) values, and comparisons were made at an *a priori* significance level of *p* ≤ 0.05. Microsoft Excel Package was used to present results graphically.

## 3. Results

**Figure 1:**
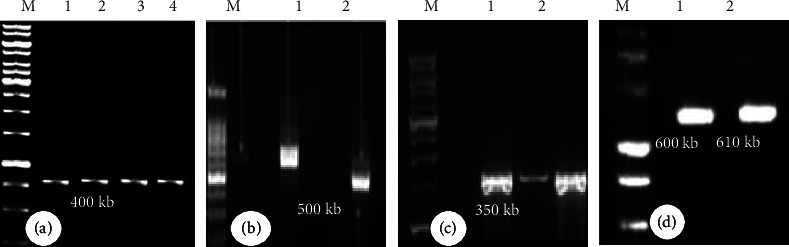
Photograph of gel electrophoresis of PCR-amplified DNA products of five wild mushroom species. (a) Lane M: 1 kb marker; lane 1–4: *A. auricula-judae* with 400 kb DNA bands; (b) lane M: 1 kb marker; lane 1-2: *M. xanthopus* with 500 kb DNA bands; (c) lane M: 1 kb marker; lane 1-2: *T. umkowaani* with 350 kb DNA bands; (d) lane M: 1 kb marker; lane 1: *T. elegans* with 600 kb DNA band; lane 2: *T. versicolor* with 610 kb DNA bands.

The specimens were identified as Trametes elegans (from Arabuko-Sokoke National Forest) and Auricularia auricula-judae, Microporus xanthopus, Termitomyces umkowaani, and Trametes versicolor (from Kakamega National Forest) based on morphological characteristics and molecular techniques, respectively (Figure 1).

The specimens were identified with the aid of mycologists and with careful observation of the physical characteristics of the mushrooms, spore prints, local knowledge, literature, taxonomy keys, and other sources. The color and shape of the cap, the color and shape of the stipe, and the size and shape of the gill were some of the morphological characteristics used to identify mushrooms. The majority of the specimens that were gathered have even gill margins, a centrally located stipe, equal stipe bases, and smooth pileal margins and surfaces. The structure of the cap, the margin and placement of the gills, the location, surface, and shape of the stipe, and the margin, shape, and surface of the pileus, among other morphological observations of the mushrooms, were also noted (Table 1). The ornamentation of the pileus and stipe surfaces, the presence or absence of an annulus on the stipe, and the presence or absence of a volva at the base of the stipe were additional characteristics that were used to define and identify the mushrooms. However, none of the specimens had any volva or annulus features. The only specimen without a stipe on its cap was Auricularia auricular-judae. The color of the upper and lower surfaces of the caps of the gathered mushrooms was noted in addition to the aforementioned variables.

The five mushrooms' amplified DNA bands have sizes ranging from 350 base pairs to 600 base pairs (Figure 1). The ITS markers can clearly distinguish between and among species of mushrooms. The examined mushrooms were identified as *A. auricula-judae, M. xanthopus, T. umkowaani, T. elegan*s, and *T. versicolor*.

### 3.1. Biofilm-Inhibitory Activities of *Auricularia auricula-judae* Extracts

**Figure 2:**
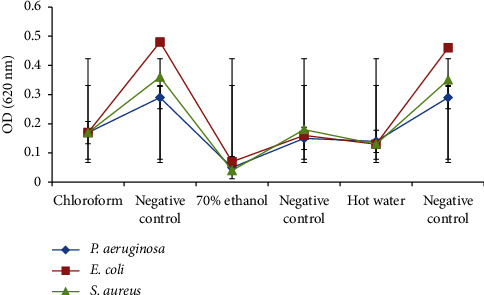
Biofilm-inhibitory activities of *A. auricula-judae* extracts against the test bacteria.

The bacterial biofilm-inhibitory activities of the three extracts of *A. auricula-judae* were detected at 50 *μ*g/mL (Figure 2). Chloroform, 70% ethanol, and hot water extracts showed statistically significant biofilm-inhibitory activities against *P. aeruginosa*, *E. coli*, and *S. aureus*, respectively (*p* ≤ 0.05). Of the three test bacteria, the biofilm formation of *S. aureus* was considerably inhibited by 70% ethanol extract.

**Figure 3:**
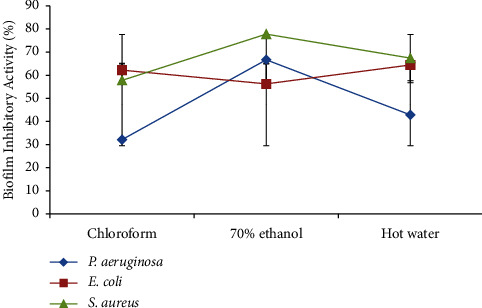
Percentage of biofilm-inhibitory activities of *A. auricula-judae* extracts against the test bacteria.

Different percentages of biofilm-inhibitory activity against the test bacteria were produced by the three *A. auricula-judae* extracts (Figure 3). The highest percentages of biofilm-inhibitory activity were demonstrated by chloroform, 70% ethanol, and hot water extracts against *E. coli* (62.22 0.02%), *S. aureus* (77.78 0.02%), and *S. aureus* (67.39 0.04%), respectively. Out of the three extracts, 70% ethanol had the strongest biofilm-inhibitory effects on *P. aeruginosa*.

At concentrations below the minimum inhibitory concentrations (if any), all *A. auricula-judae* extracts were evaluated for their antibacterial effectiveness against test microorganisms (Table 3). The chloroform extract had antibacterial effects on *E. coli*. Extracts produced with hot water and 70% ethanol, however, exhibited no antibacterial action at the investigated concentration.

### 3.2. Biofilm-Inhibitory Activities of *Microporus xanthopus* Extracts

**Figure 4:**
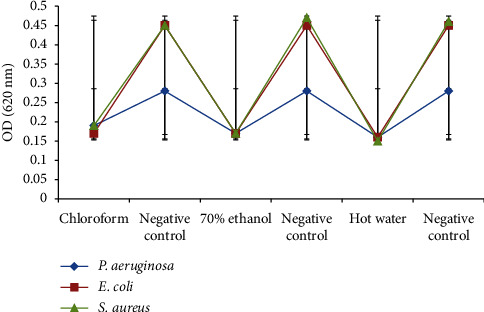
Biofilm-inhibitory activities of *M. xanthopus* extracts against the test bacteria.

All *M. xanthopus* extracts (250 g/mL) exhibited strong biofilm-inhibitory effects against the test microorganisms (Figure 4). Chloroform extract showed statistically significant biofilm-inhibitory activity against each test bacterium (*P. aeruginosa*, *E. coli*, and *S. aureus*) and its respective control bacterium, respectively (*p* ≤ 0.05). Likewise, the 70% ethanol extract resulted in statistically significant biofilm-inhibitory activity against *P. aeruginosa*, *E. coli*, and *S. aureus*, respectively (*p* ≤ 0.05). The hot water extract also yielded statistically significant biofilm-inhibitory activity against *P. aeruginosa*, *E. coli*, and *S. aureus*, respectively (*p* ≤ 0.05).

**Figure 5:**
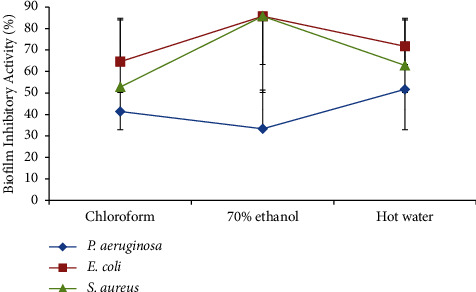
Percentage of biofilm-inhibitory activities of *M. xanthopus* extracts against the test bacteria.

*M. xanthopus* extracts' percentage of biofilm-inhibitory activities was evaluated in a concentration-dependent manner (Figure 5). When compared to the control bacteria, the test bacteria significantly reduced the production of biofilms. Hot water and chloroform extracts reduced the biofilm-formation activity of *P. aeruginosa* (51.72 ± 0.01%) and *E. coli* (64.58 ± 0.03%), respectively. The 70% ethanol extract had significant biofilm-inhibitory effects against *E. coli* and *S. aureus* (85.71 0.01%) but had little impact on *P. aeruginosa* (33.33 0.03%) in terms of biofilm formation. All of the extracts had minimal biofilm-inhibitory effects against *P. aeruginosa*.

The effects of *M. xanthopus* extracts on the development of planktonic cells were assessed in comparison to the test bacterium. Only the planktonic cell of *S. aureus* was suppressed by all the extracts. However, *P. aeruginosa* and *E. coli* planktonic cells' proliferation has not been inhibited by the extracts (Table 4).

### 3.3. Biofilm-Inhibitory Activities of *Termitomyces umkowaani* Extracts

**Figure 6:**
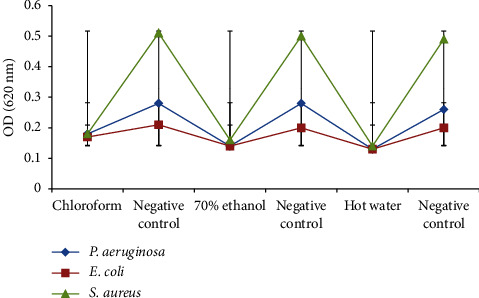
Biofilm-inhibitory activities of *T. umkowaani* extracts against the test bacteria.

At a concentration of 250 g/mL, *T. umkowaani* extracts showed biofilm-inhibitory effects against *P. aeruginosa, E. coli,* and *S. aureus* (Figure 6). Inhibitory biofilm activity against all of the test microorganisms was lowest in the chloroform extract. All extracts resulted in limited biofilm-inhibitory activities against *P. aeruginosa* and *E. coli* as compared to *S. aureus*. Chloroform, 70% ethanol, and hot water extracts showed statistically significant biofilm-inhibitory activities against *S. aureus* (*p* ≤ 0.05).

**Figure 7:**
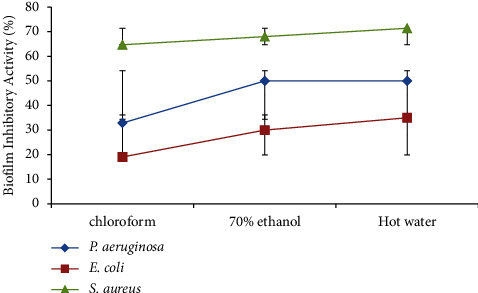
Percentage of biofilm-inhibitory activities of *T. umkowaani* extracts against the test bacteria.

The percentage of biofilm-inhibitory activities of *T. umkowaani* extracts was evaluated against the test bacteria (Figure 7). All extracts showed a weak percentage of antibiofilm-formation activities against *E. coli*. The best biofilm-inhibitory activities were observed with chloroform (64.71 ± 0.01%), 70% ethanol (68 ± 0.01%), and hot water (71.43 ± 0.02%) extracts against the *S. aureus*. Of the three extracts, the hot water extract exhibited a better percentage of biofilm-inhibitory activities against all the test bacteria. The 70% ethanol and hot water extracts significantly limited (as much as 50%) the biofilm formation of *P. aeruginosa*.

The chloroform extract resulted in growth inhibition of *E. coli* but not *P. aeruginosa* and *S. aureus*. Yet, the 70% ethanol and hot water extracts did not result in any antibacterial activities in all the test bacteria (Table 5).

### 3.4. Biofilm-Inhibitory Activities of *Trametes elegans* Extracts

**Figure 8:**
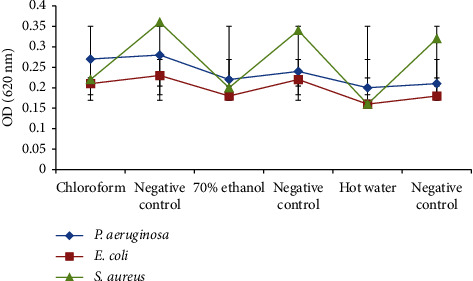
Biofilm-inhibitory activities of *T. elegans* extracts against the test bacteria.

The biofilm-inhibitory activity of *T. elegans* extracts (250 *μ*g/mL) yielded statistically significant effects between the test and the negative control bacteria (*p* ≤ 0.05). All extracts of *T. elegans* demonstrated weak biofilm-inhibitory activities against *P. aeruginosa* and *E. coli* (Figure 8). However, the chloroform, 70% ethanol, and hot water extracts showed statistically significant biofilm-inhibitory activities against *S. aureus* (*p* ≤ 0.05). The biofilm-inhibitory activity of the extracts was effective against *S. aureus*.

**Figure 9:**
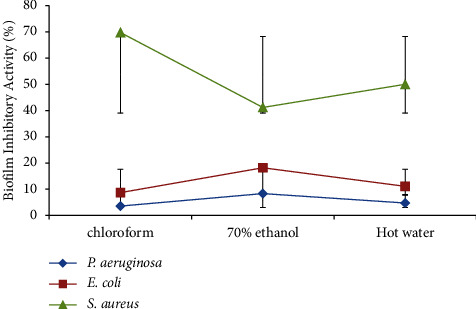
Percentage of biofilm-inhibitory activities of *T. elegans* extracts against the test bacteria.

The percentage of biofilm-inhibitory activities of *T. elegans* extracts was very weak against all the test bacteria (Figure 9). All the extracts showed a very weak percentage of biofilm-inhibitory activities against *P. aeruginosa* and *E. coli*. Comparatively speaking, the chloroform extract yielded the best percentage of biofilm-inhibitory activity against *S. aureus* (69.75 ± 0.01%) as compared to the hot water (50 ± 0.02%) and 70% ethanol (41.18 ± 0.01%) extracts.

The effects of the extracts of *T. elegans* on the antibacterial and the biofilm-inhibitory activities were determined against all the test bacteria (Table 6). Although the chloroform extract showed antibacterial activity against *E. coli*, there was no such effect on *P. aeruginosa* and *S. aureus*. On the other hand, the 70% ethanol and hot water extracts demonstrated limited biofilm-inhibitory activities against all the test bacteria without any effect on their growth.

### 3.5. Biofilm-Inhibitory Activities of *Trametes versicolor* Extracts

**Figure 10:**
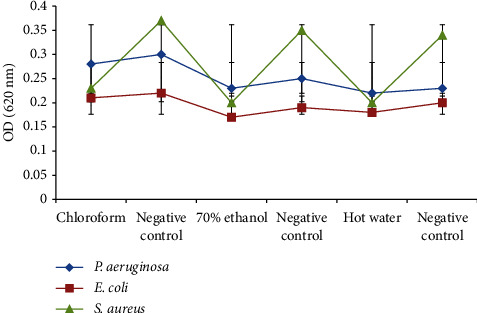
Biofilm-inhibitory activities of *T. versicolor* extracts against the test bacteria.

The biofilm-inhibitory activities of the extracts of *T. versicolor* (250 *μ*g/mL) against test and control bacteria yielded no statistically significant results (*p* > 0.05) (Figure 10). All the extracts resulted in a weak percentage of biofilm-inhibitory activities against all the test bacteria.

**Figure 11:**
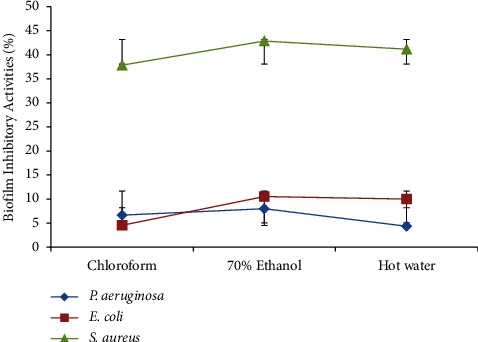
Percentage of biofilm-inhibitory activities of *T. versicolor* extracts against the test bacteria.

The percentage of biofilm-inhibitory activities of *T. versicolor* extracts against the test microorganisms was investigated (Figure 11). A very small percentage of biofilm-inhibitory activity against *P. aeruginosa and E. coli* was produced by all three extracts. However, all extracts showed the highest percentage of biofilm-inhibitory activities against *S. aureus*. Hot water (41.18 ± 0.01%) and 70% ethanol (42.86 ± 0.01%) extracts exhibited the lowest and the highest percentage of biofilm-inhibitory activities against *S. aureus*. Besides, the 70% ethanol extract showed the highest percentage of biofilm-inhibitory activity against *S. aureus* (42.86 ± 0.01%) and lowest against *E. coli* (10.53 ± 0.01%).

*P. aeruginosa* and *S. aureus* planktonic cells did not experience any growth suppression from the chloroform extract, despite the fact that *E. coli* did (Table 7). Both a 70% ethanol extract and a hot water extract had a discernible inhibitory effect on the growth of planktonic cells when used against *S. aureus* and *P. aeruginosa*.

## 4. Discussion

The results of the current investigation demonstrated that *A. auricula-judae* and *M. xanthopus* extract in chloroform, 70% ethanol, and hot water had effective biofilm-inhibitory properties. The ability of the extracts to inhibit the biofilm-formation sites (such as cyclic diguanylate) of the test bacteria may be one of the many potential explanations for these promising biofilm-inhibitory capabilities. Cyclic diguanylate starts the synthesis of bis-(3′-5′)-cyclic dimeric guanosine monophosphate, which makes it possible to produce sticky compounds connected to the biofilm development process continuously [34]. The extracts may also be able to considerably repress a number of genes and prevent the production of sticky compounds necessary for the processes of quorum sensing and biofilm formation [35, 36].

The test bacteria's ability to form biofilms was significantly reduced by the biofilm-inhibitory effects of *A. auricula-judae* and *M. xanthous* extracts. However, the extracts displayed a range of biofilm-formation inhibitory effects against *E. coli, P. aeruginosa*, and *S. aureus. P. aeruginosa's* capacity to form biofilms was diminished by hot water extracts of *A. auricula-judae* and *M. xanthopus*. Similarly, all of the *A. auricula-judae* and *M. xanthopus* extracts showed superior biofilm-inhibitory properties against S. aureus but were less efficient in preventing *P. aeruginosa* from forming biofilms. This might be attributed to and explained by the two bacteria's contrasting efflux pumps for the extracts and their distinct affinities for certain signaling molecules [37]. Moreover, the recovered bioactive chemicals cannot enter the bacterial cells because of the structure of the polysaccharides that surround them [38].

Extracts of *A. auricula-judae* and *M. xanthous* prevented the test bacteria from forming biofilms without impairing the growth of their planktonic cells. These results show that the extracts prevented or reduced the test bacteria's ability to form biofilms at a concentration of 50 g/mL, which is much lower than the minimum inhibitory concentration values noted in our earlier studies [39, 40]. The receptor proteins and molecules involved in the quorum-sensing pathway may have had their activity reduced by these extracts. According to certain research, extract concentrations below MIC values do not necessarily kill the test bacteria; instead, they have an alternative method of action that prevents the adhesion process [31]. Yet, this is not always the case. An investigation into oral biofilms revealed that extracts of Lentinula edodes at and below the MIC values did not significantly slow down the biofilm-formation process [41].

Even though all of the *A. auricula-judae* and *M. xanthopus* extracts only had modest antimicrobial effects on *P. aeruginosa*, they had significant biofilm-inhibitory effects. According to these results, extracts with good antimicrobial activity do not necessarily have good biofilm suppressive activities [42]. This may be explained by the fact that biofilms have unique phenotypic characteristics that are not present in their planktonic cousins. For example, biofilm-forming bacteria boost rates of genetic exchange among other biofilm occupants and boost resistance to environmental variables such antibiotics, detergents, UV exposure, dehydration, salt, and phagocytosis [42–44].

The biofilm-forming ability of *S. aureus* was severely reduced by all *T. umkowaani* extracts. These extracts' capacity to prevent the development of the enzymes and proteins necessary for the creation of extracellular polymeric compounds may explain their ability to suppress biofilm-inhibitory effects against S. aureus. These results are consistent with those of earlier studies that looked at the antibiofilm properties of aqueous extracts against *Streptococcus mutans* [45]. This may be because these extracts inhibit DNA gyrase, which is necessary for DNA synthesis. Similar to this, the extracts' abilities to suppress biofilm formation could be brought on by the bacterium's blockage of quorum sensing. Extracts' antiquorum-sensing abilities have the ability to quickly alter the gene expression patterns of a bacterial population in response to population density [31].

*S. aureus*'s ability to form biofilms was markedly reduced by a hot water extract of *T. elegans*. This may be related to the extracts' detrimental effects on the bacterial motility and extracellular polymer synthesis during the biofilm development stages [46]. Additionally, the extracts may be able to obstruct the early phases of biofilm development by preventing the production of extracellular polysaccharide molecules and the initial adhesion of the bacterial cells to the surfaces [47–51].

Comparatively to the standard bacterial strain of *S. aureus*, all *T. versicolor* extracts showed poor biofilm-inhibitory effects against the clinical isolates of *P. aeruginosa* and *E. coli*. The observed weak antibiofilm effects of the extracts on the clinical isolates, inherent resistance to antimicrobial agents and some genetically controlled processes, may be responsible for the observed weak biofilm-inhibitory activities [52, 53]. Strong biofilm development, as seen in the *P. aeruginosa* isolates, is a crucial component in the pathogenesis of many bacterial illnesses as well as their survival and flourishing in a variety of habitats. In order to establish a host, increase their population, and, most critically, spread disease, bacterial pathogens may rely on the emergence of biofilms [52, 54, 55].

## 5. Conclusions

The results suggest that the mushroom extracts would be a good option for biofilm-inhibitory properties. In a concentration-dependent pattern, all mushroom extracts demonstrated considerable biofilm-inhibitory efficacy against the test organisms. All extracts were more efficient against S. aureus at inhibiting biofilms. The intriguing potential of the extracts to inhibit the bacteria's biofilm-formation processes might lead to the discovery of new chemicals that might find use in clinical settings. Researchers may be prompted to hunt for new substances with potential for use in medicine by the extracts' promising capacity to significantly block the test bacteria's biofilm-formation activities. To pinpoint the active extract ingredients that are responsible for the extracts' biofilm-inhibiting properties, more research is required. It is vital to identify these bioactive substances and explain how they work in order to exert their antibiofilm effects.

### 5.1. Future Prospective of the Study

The pharmaceutical and medical biotechnology sectors, as well as human health, will be significantly impacted by the research findings discussed in this paper. Therefore, it is crucial to utilize wild mushrooms as food and a unique source of antibacterial and antibiofilm chemicals. With the aid of these bioactive molecules, the search for novel compounds to combat the troublesome characteristics of biofilm-forming and drug-resistant bacteria might also enter a new era.

## Figures and Tables

**Table 1:** Morphological characters and keys used for the identification of the wild mushrooms.

Mushrooms species	Cap structure	Location of stipe	Pileus margin	Pileal shape	Pileal surface	Annulus (veil/ring)	Volva (cup)	Stipe surface	Stipe base/shape	Gill margin	Gill attachment
*Auricularia auricula-judae*	N/A	N/A	Incurved	Ear shaped	N/A	—	—	N/A	No stipe	N/A	N/A
*Microporus xanthopus*	Half funnel	N/A	Smooth	Hemispherical	Smooth	—	—	N/A	Substipitate	N/A	Sinuate
*Termitomyces umkowaani*	Umbonate	Central	Smooth	Knobbed	Smooth	—	—	Smooth	Swollen	N/A	Adnexed
*Trametes elegans*	Funnel-like	N/A	Smooth	N/A	Rough	—	—	N/A	Nonstipe	N/A	Adnate
*Trametes versicolor*	N/A	N/A	N/A	N/A	N/A	—	—	N/A	Substipitate	N/A	Free

*Note*. N/A: not applicable, (—) denotes the absence of annulus and volva.

**Table 2:** Primers used to amplify specific genes of the five mushroom species.

Mushrooms	Primers (forward and reverse)	Required genes
*Auricularia auricula-judae*	ITS1-F (5′-CTT GGT CAT TTA GAG GAA GTA A-3′)	*RPB2*
	ITS5 (5′-GGA AGT AAA AGT CGT AAC AAGG-3′)	

*Microporus xanthopus*	ITS1-F (5′-CTT GGT CAT TTA GAG GAA GTA A-3′)	*ITS nrDNA*
	ITS4 (5′-TCC TCC GCT TAT TGA TAT GC-3′)	

*Termitomyces umkowaani*	ITS1-F (5′-CTT GGT CAT TTA GAG GAA GTA A-3′)	*LSU rDNA*
	ITS4 (5′-TCC TCC GCT TAT TGA TAT GC-3′)	

*Trametes elegans*	ITS1-F (5′-CTT GGT CAT TTA GAG GAA GTA A-3′)	*TEF1α*
	ITS4 (5′-TCC TCC GCT TAT TGA TAT GC-3′)	

*Trametes versicolor*	ITS1-F (5′-CTT GGT CAT TTA GAG GAA GTA A-3′)	*TEF1α*
	ITS4 (5′-TCC TCC GCT TAT TGA TAT GC-3′)	

*Note*. *RPB2*: RNA polymerase II largest subunit gene; *TEF1α*: translation elongation factor 1-alpha; LSU rRNA: large subunit ribosomal RNA; *ITS nrDNA*: internal transcribed spacer nuclear ribosomal DNA.

**Table 3:** Effect of *A. auricula-judae* extracts on the growth of planktonic bacterial cells.

Test bacteria	Optical density of the test and the negative control bacteria at 620 nm					
	Chloroform	NC	70% ethanol	NC	Hot water	NC
*P. aeruginosa*	0.20 ± 0.01	0.20 ± 0.01	0.10 ± 0.02	0.10 ± 0.01	0.20 ± 0.02	0.20 ± 0.02
*E. coli*	**0.10** **±** **0.02**	**0.20** **±** **0.01**	0.20 ± 0.02	0.20 ± 0.01	0.10 ± 0.01	0.10 ± 0.02
*S. aureus*	0.20 ± 0.01	0.20 ± 0.02	0.20 ± 0.01	0.20 ± 0.02	0.10 ± 0.02	0.10 ± 0.01

NC: negative control; all extracts were tested at 62.5 *μ*g/mL. The significance of the bold values is to indicate that the chloroform extract has shown promising results beyond the negative control.

**Table 4:** Effect of *M. xanthopus* extracts on the growth of planktonic bacterial cells.

Test bacteria	Optical density of the test and the negative control bacteria at 620 nm					
	Chloroform	NC	70% ethanol	NC	Hot water	NC
*P. aeruginosa*	0.10 ± 0.01	0.10 ± 0.02	0.10 ± 0.02	0.10 ± 0.01	0.20 ± 0.01	0.20 ± 0.01
*E. coli*	0.10 ± 0.01	0.10 ± 0.01	0.20 ± 0.02	0.20 ± 0.02	0.20 ± 0.01	0.20 ± 0.02
*S. aureus*	**0.10** **±** **0.01**	**0.20** **±** **0.01**	**0.20** **±** **0.02**	**0.30** **±** **0.01**	**0.20** **±** **0.02**	**0.30** **±** **0.02**

NC: negative control; all extracts were tested at 250 *μ*g/mL. The significance of the bold values is to indicate that all the extracts have shown promising results against *S. aureus* even beyond the negative control.

**Table 5:** Effect of *T. umkowaani* extracts on the growth of planktonic bacterial cells.

Test bacteria	Optical density of the test and the negative control bacteria at 620 nm					
	Chloroform	NC	70% ethanol	NC	Hot water	NC
*P. aeruginosa*	0.20 ± 0.02	0.20 ± 0.01	0.30 ± 0.01	0.30 ± 0.00	0.10 ± 0.01	0.10 ± 0.02
*E. coli*	**0.10** **±** **0.01**	**0.20** **±** **0.00**	0.20 ± 0.02	0.20 ± 0.01	0.10 ± 0.00	0.10 ± 0.01
*S. aureus*	0.20 ± 0.00	0.20 ± 0.00	0.10 ± 0.01	0.10 ± 0.00	0.20 ± 0.02	0.20 ± 0.00

NC: negative control; all the extracts were tested at 250 *μ*g/mL. The significance of the bold values is to indicate that the chloroform extract has shown promising results against *E. coli* even beyond the negative control.

**Table 6:** Effect of *T. elegans* extracts on the growth of planktonic bacterial cells.

Test bacteria	Optical density of the test and the negative control bacteria at 620 nm					
	Chloroform	NC	70% ethanol	NC	Hot water	NC
*P. aeruginosa*	0.20 ± 0.00	0.20 ± 0.00	0.10 ± 0.02	0.10 ± 0.01	0.10 ± 0.00	0.10 ± 0.02
*E. coli*	**0.20** **±** **0.02**	**0.30** **±** **0.01**	0.20 ± 0.02	0.20 ± 0.02	0.20 ± 0.01	0.20 ± 0.01
*S. aureus*	0.30 ± 0.01	0.30 ± 0.00	0.10 ± 0.01	0.20 ± 0.01	0.20 ± 0.02	0.20 ± 0.00

NC: negative control; all extracts were tested at 250 *μ*g/mL. The significance of the bold values is to indicate that the chloroform extract has shown promising results against *E. coli* even beyond the negative control.

**Table 7:** Effect of *T. versicolor* extracts on the growth of planktonic bacterial cells.

Bacteria	Optical density of the test and the negative control bacteria at 620 nm					
	Chloroform	NC	70% ethanol	NC	Hot water	NC
*P. aeruginosa*	0.20 ± 0.00	0.20 ± 0.00	0.10 ± 0.01	0.10 ± 0.00	**0.10** **±** **0.02**	**0.20** **±** **0.00**
*E. coli*	**0.20** **±** **0.01**	**0.30** **±** **0.00**	0.20 ± 0.01	0.20 ± 0.00	0.20 ± 0.00	0.20 ± 0.00
*S. aureus*	0.30 ± 0.01	0.30 ± 0.01	**0.10** **±** **0.02**	**0.20** **±** **0.00**	**0.20** **±** **0.01**	**0.30** **±** **0.00**

NC: negative control; all extracts were tested at 250 *μ*g/mL. The significance of the bold values is to indicate that all extracts have shown promising results against all bacteria even beyond the negative control.

## Data Availability

The data used to support the findings of this study are available from the corresponding author upon request.
